# Methylmercury: Grandjean et al. Respond

**DOI:** 10.1289/ehp.1103580R

**Published:** 2011-07-01

**Authors:** Philippe Grandjean, Hiroshi Satoh, Katsuyuki Murata, Komyo Eto

**Affiliations:** Department of Environmental Health, Harvard School of Public Health, Boston, Massachusetts, E-mail: pgrand@hsph.harvard.edu; Department of Environmental Health Sciences, Tohoku University Graduate School of Medicine, Sendai, Japan; Division of Environmental Health Sciences, Akita University, Akita, Japan; National Institute for Minamata Disease, Minamata, Japan

We thank Tsuda et al. for sharing their views. Along with their previous publications on Minamata disease, we find their comments useful as a complement to our brief historical review of the mass poisonings in Japan and associated events (Grandjean et al. 2010). However, the specific issues raised in their letter do not affect our conclusions on the research implications of the history of methylmercury science.

## References

[r1] Grandjean P, Satoh H, Murata K, Eto K. (2010). Adverse effects of methylmercury: envionmental health research implications.. Environ Health Perspect.

